# Supplementary material for: Content-rich biological network constructed by mining PubMed abstracts
Source: BMC Bioinformatics. 2004 Oct 8;5:147. doi: 10.1186/1471-2105-5-147 (PMC528731; doi:10.1186/1471-2105-5-147)
Supplement: Additional File 2 — The original results of the above study (non-essential files are deleted to keep the file size under the limit set by BMC bioinformatics). [file 1471-2105-5-147-S2.bz2 › chilibotAdditionalFile2/dip10/365ID8663044E6038/html/FGFR2_FGF7.html]

 


 **FGFR2** and **FGF7** 
  
Found 200 abstracts in PubMed, retrieved 10.  
 

 What does Google say? 
 PDF only 
| .edu only 

---

**Interactive relationship** (e.g. stimulation, inhibition, etc)

**Neutral relationship**- KGF  [ **FGF7** ]  upregulated EC KGFR  [ **FGFR2** ]  mRNA expression, predominately in the crypt and lower portion of the villus.  Ref: 12757114 JPEN J Parenter Enteral Nutr< MedlineTA>JPEN J Parenter Enteral Nutr,
- Exposure to both UVB 30 150 mJ cm 2 and CUH 200 microM of NIH3T3 KGFR  [ **FGFR2** ]  KGF  [ **FGF7** ]  receptors transfectants caused a rapid tyrosine phosphorylation and activation of KGFR  [ **FGFR2** ]  similar to that induced by KGF  [ **FGF7** ] , and internalization of the activated receptor.  Ref: 12717419 Oncogene, 2003
- The relative mitogenic efficacy of basic FGF, acidic FGF, or KGF  [ **FGF7** ]  was in correlation with their affinities for the KGF  [ **FGF7** ]  receptor KGFR  [ **FGFR2** ] .  Ref: 12761878 J Cell Biochem, 2003
- Taken together our results demonstrate that exposure to UVB and to oxidant stimuli induces a rapid intracellular production of ROS, which in turn are capable of triggering KGFR  [ **FGFR2** ]  activation and internalization, similar to those induced by KGF  [ **FGF7** ] .  Ref: 12717419 Oncogene, 2003
- FGF1,  **FGF7** , FGF9, FGF10, FGF11, and FGF18 were also expressed in the pancreas at varying time points and levels, as well as FGF receptor  [ **FGFR2** ]  forms FGFR1b, FGFR1c, FGFR2b, FGFR2c, FGFR3b, and FGFR4.  Ref: 12666204 Dev Dyn, 2003

**Non-interactive relationship** (e.g. studied together, co-existance, homology, etc.)

- The alpha5 integrin, keratinocyte growth factor  [ **FGF7** ]  receptor  [ **FGFR2** ]  and cyclin D1 mRNA levels were higher compared to the controls as early as 2 h after ethanol and 30 min after acetone treatment of the cells.  Ref: 12720008 Arch Dermatol Res< MedlineTA>Arch Dermatol Res, 2003
- Epithelial KGF  [ **FGF7** ]  receptor KGFR  [ **FGFR2** ]  mRNA expression was studied using real time reverse transcriptase polymerase chain reaction RT PCR.  Ref: 12757114 JPEN J Parenter Enteral Nutr< MedlineTA>JPEN J Parenter Enteral Nutr, 2003
- The effects of ethanol and acetone on the mRNA levels of genes characteristic for proliferating keratinocytes such as alpha5 integrin, keratinocyte growth factor  [ **FGF7** ]  receptor  [ **FGFR2** ]  and cyclin D1 were studied by reverse transcriptase polymerase chain reaction.  Ref: 12720008 Arch Dermatol Res< MedlineTA>Arch Dermatol Res, 2003
- Our results support the notion that the paracrine KGF  [ **FGF7** ]  KGFR  [ **FGFR2** ]  system in the skin can act in concert with the autocrine vitamin D system in keratinocytes to promote keratinocyte proliferation and survival under situations of stress and injury.  Ref: 12761878 J Cell Biochem, 2003
- P63a mutations lead to aberrant splicing of keratinocyte growth factor  [ **FGF7** ]  receptor  [ **FGFR2** ]  in the Hay Wells Syndrome.  Ref: 12692135 J Biol Chem< MedlineTA>J Biol Chem, 2003
- Treatment with 5 OH 2 D 3 increased the levels of KGFR  [ **FGFR2** ]  in the presence but not in the absence of KGF  [ **FGF7** ] , probably due to inhibition of ligand induced receptor degradation.  Ref: 12761878 J Cell Biochem, 2003
- UVB induced activation and internalization of keratinocyte growth factor  [ **FGF7** ]  receptor  [ **FGFR2** ] .  Ref: 12717419 Oncogene, 2003
- The gene expression of HGF and keratinocyte growth factor  [ **FGF7** ]  KGF  [ **FGF7** ]  in gingival connective tissue, and c met, HGFA and KGF  [ **FGF7** ]  receptor KGFR  [ **FGFR2** ]  in gingival epithelial tissues was monitored using RT PCR.  Ref: 12613501 J Oral Sci, 2002
- Vitamin D enhances mitogenesis mediated by keratinocyte growth factor  [ **FGF7** ]  receptor  [ **FGFR2** ]  in keratinocytes.  Ref: 12761878 J Cell Biochem, 2003
